# Deep-sea crustacean trawling fisheries in Portugal: quantification of effort and assessment of landings per unit effort using a Vessel Monitoring System (VMS)

**DOI:** 10.1038/srep40795

**Published:** 2017-01-18

**Authors:** Juan Bueno-Pardo, Sofia P. Ramalho, Ana García-Alegre, Mariana Morgado, Rui P. Vieira, Marina R. Cunha, Henrique Queiroga

**Affiliations:** 1 Departamento de Biologia & Centro de Estudos do Ambiente e do Mar (CESAM), Universidade de Aveiro, Aveiro, 3810-193, Portugal; 2 Ghent University, Marine Biology Research Group, Ghent, Krijgslaan 281 S8, 9000, Belgium; 3 Graduate School of the National Oceanography Centre Southampton, University of Southampton, Waterfront Campus, European Way, Southampton SO14 3ZH, United Kingdom

## Abstract

Mapping and quantifying bottom trawling fishing pressure on the seafloor is pivotal to understand its effects on deep-sea benthic habitats. Using data from the Vessel Monitoring System of crustacean trawlers along the Portuguese margin, we have identified the most exploited areas and characterized the most targeted habitats and water depths. We estimated a total trawling effort of 69596, 66766, and 63427 h y^−1^ for the years 2012, 2013, and 2014 respectively which, considering the total landings estimated for this gear, yield values of 20.76, 21.06, and 19.11 kg of landed fish per trawled hour. The main trawling pressure is exerted in the South and Southwest Portuguese margins, on muddy and muddy-sand bottoms between 200 and 700 m water depths, while in the North and Central-West coasts a minor effort, at shallower waters and across a wider range of habitats, is also applied. The most landed species are crustaceans such as rose shrimp and Norway lobster, although this varies importantly between the different regions of Portugal, being fish and cephalopods the main captures in the Northern ports. We discuss the consequences of trawling for the impacted communities as well as the characteristics of the commercialization of these captures in Portugal.

Global deep-water fisheries (>200 m) have increased in importance since the 1950’s due to the development of more powerful fishing fleets and the evolution of specific techniques and gears. Since then, as shallower and more accessible stocks became depleted, a shift towards deeper areas has been reported^1^. Of the *métiers* used in the deep-sea, bottom trawling is among the most destructive to the environment. Direct consequences include the modification of the seabed by physical destruction, the removal of benthic communities and the direct mortality of individuals, while indirect effects include enhanced mortality of damaged individuals, changes in the sediment biogeochemistry and alterations in the food web structure^2^,^3^,^4^,^5^,^6^. The characteristics of the gear, including size, weight, and the frequency of the activity, are key factors determining the intensity of the perturbations on the ecosystem, whereas considering the characteristics of the habitats and fauna where trawling occurs are pivotal factors to determine the ecosystem capacity to resist and recover from perturbations^7^,^8^.

The Portuguese trawl fleet is currently composed by 128 vessels, of which 25 have licenses for fishing crustaceans and 103 for fin-fishes. Five Spanish crustacean trawlers also operate in Portuguese waters as a result of an agreement between both countries^9^. The division of the fleet in two components can be made based on target species, where crustacean trawlers target shrimps and Norway lobster (*Nephrops norvegicus*) with a mesh size of 55–59 mm and 70 mm respectively, and fin-fishes with a 65–69 mm mesh sized-net^10^. A characterization of the landing profiles and fleet components of these *métiers* has been made by Campos, *et al*.^11^, finding that the crustacean fleet essentially targets Norway lobster, rose shrimp (*Parapenaeus longirostris*) and red shrimp (*Aristeus antennatus*). These authors also reported fish species caught by crustacean trawlers and their proportions in the landings, namely the hake (*Merluccius merluccius*), horse mackerel (*Trachurus trachurus*), blue whiting (*Micromesistius poutassou*) and cephalopods of the family Octopodidade (e.g. *Octopus vulgaris*). The number of licensed fishermen for trawling in Portugal in 2014 was 1241, which represents 7.4% of the fishing licenses of Portugal^12^. The economic importance of crustacean trawling for the Portuguese fisheries is remarkable given the high prices of some of the target species. For instance, the landings captured by crustacean trawlers in 2013 and 2014 accounted for 16.5 and 15.2 10^6^ € respectively^12^.

Despite its economic importance, the environmental impact of trawling has been demonstrated to be unsustainable in the long term^13^,^14^. The commercial exploitation of the deep-sea must be monitored and managed with attention to the nature and selectivity of the deployed gears, the vulnerability of the exploited habitats and the biology of both target and non-target species (e.g. many of the deep-sea species have a slow recoverability rate due to their k-selected life history traits such as slow growth and long lifespans)^15^,^16^,^17^. In this sense, this work aims to provide an accurate mapping of the distribution of trawling effort, as well as new insights into the situation of crustacean trawling in the Portuguese continental margin, with special attention to the quantification of trawling effort imposed in the different deep-sea habitats. To do this, we first map the fishing pressure exerted by crustacean trawlers during 2012, 2013, and 2014 using data from a vessel monitoring system (VMS), identifying the areas with higher trawling intensity, and quantifying the fishing effort in cells of 0.1 decimal degrees. Subsequently, we characterize the regional patterns of exploited depths, habitats and species landed, providing an estimation of the landings per unit effort (LPUE) for this *métier* along the Portuguese margin.

## Results

### Fishing effort and its geographic distribution

**Figure 1:**
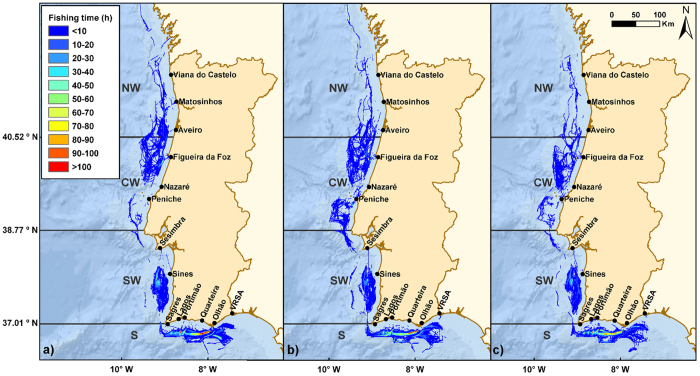
Trawling effort distribution by the whole crustacean trawling fleet in Portuguese waters during (**a**) 2012 (69596 h y^−1^), (**b**) 2013 (66766 h y^−1^), and (**c**) 2014 (63427 h y^−1^). Coordinates system used: WGS 1984 UTM Zone 29 N. Service Layer Credits: General Bathymetric Chart of the Oceans (GEBCO). Map created using ArcGIS v.10.2, https://www.arcgis.com/.

The total trawling effort estimated for crustacean trawlers in Portuguese waters was 69596 h y^−1^ in 2012; 64198 h y^−1^ in 2013; and 60988 h y^−1^ in 2014 (Fig. 1). For each vessel, these results correspond to an annual trawling effort of 2676 h y^−1^ (2012); 2568 h y^−1^ (2013); and 2439 h y^−1^ (2014). The average number of fishing trips performed by each vessel per year were 120.2, 115.9, and 112.6, with durations of 38.9, 40.9, and 39.6 hours, while the ratio between fishing time and time at sea was 0.538, 0.494, and 0.503 respectively. During the three years of study, the total area of the Portuguese margin affected by crustacean trawling remained relatively constant, with a total of 12262, 13815, and 11349 km^2^ for 2012, 2013 and 2014. The distribution of the effort through the different geographic regions shows a particular concentration of trawling in the South and South-West margins of Portugal (Fig. 1), averaging 11.13 and 4.96 trawling hours per year per impacted cell respectively, while in the North and Central-West regions, impacted-cells were trawled on average during 1.13 and 1.96 hours per year (Table 1). Translated to the percentage of area of the geographic cells affected by trawling, these impact indices correspond to average values of 50% in the North-West; 86% in the Central-West; 219% in the South-West; and 492% in the South (a value of 100% indicates that the accumulated area of each cell trawled in 1 year equals the total area of the cell; see methods). These impact indices have remarkably decreased during the three years of study in all the regions with exception of the South-West (Table 1).

Table 2 shows the trawling effort corresponding (trawling hours y^−1^) to the different ports and regions of Portugal. The most important ports in terms of reception of trawling effort were Portimão (39.3%), Olhão (23.1%), and Sines (12.3%). Considering the distribution of the effort through the regions, the southern ports are the most important receptors of trawling time (average 64.8%), although its importance in relation to the remaining Portuguese ports decreased during the three years of study. The same happened in the North-West region, while the Central and South-West divisions are more variable and seem to have increased in importance (Table 2). Portuguese trawlers also landed fish in Spain, which explains the mismatch between the total trawling effort exerted and the total trawling effort delivered to the Portuguese ports (shown in Table 2).

### Landings and Landings per Unit Effort

Total landings at Portuguese ports from crustacean trawlers were estimated as 1361, 1127, and 992 t y^−1^ for 2012, 2013, and 2014 respectively (Table 3). Considering the total trawling effort delivered to the Portuguese ports (see above), these values yielded LPUE values of 20.76, 21.06, and 19.11 kg h^−1^ in 2012, 2013, and 2014 respectively. The most important species landed by crustacean trawlers were the rose shrimp, dominating the landings during the three years of study with 669, 391, and 416 t y^−1^ (41%), followed by *N. norvegicus* (12%), *M. merluccius* (10%), and *O. vulgaris* (9%). The most important port in terms of landings for crustacean trawlers in Portugal was Vila Real de Santo António (VRSA), with an average 62.24% of the total landings (Table 3). None of the other ports received more than 10% of the Portuguese landings by crustacean trawlers in any of the years evaluated.

### Target species and geographic regions in relation to deep-sea habitats

**Figure 2:**
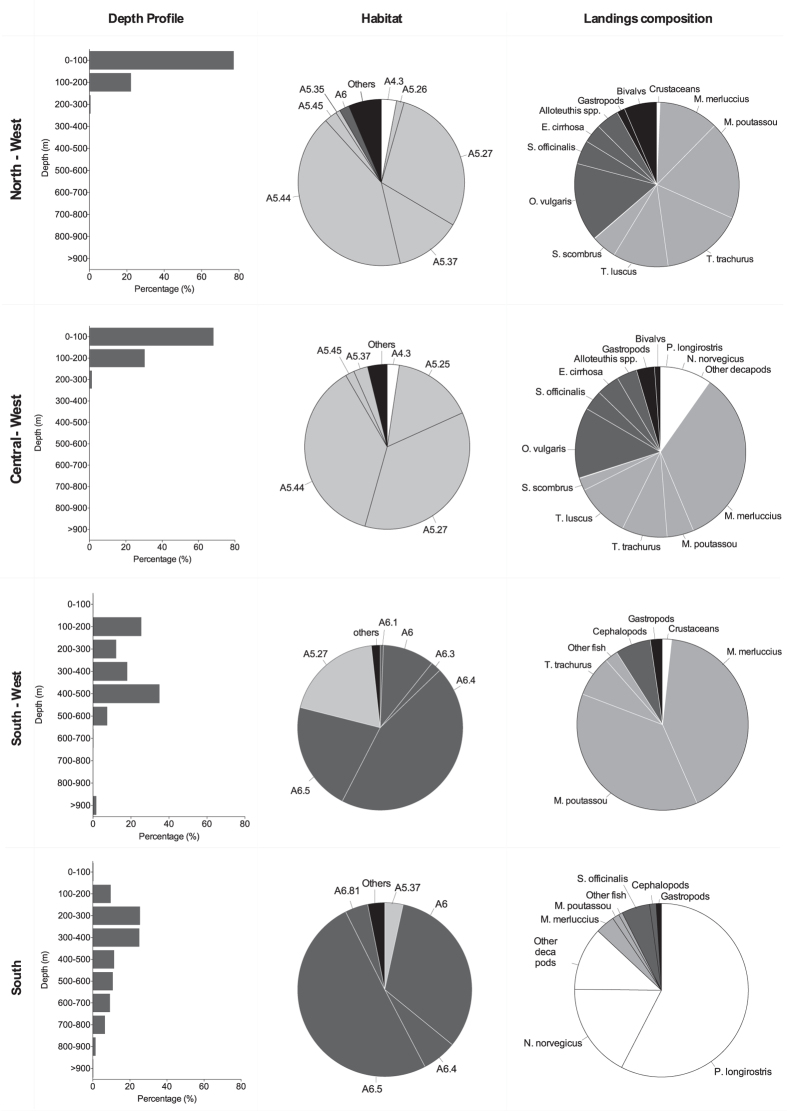
Distribution by geographic regions of the target depths, habitats and landed species by crustacean trawlers during the period of the study. Habitats represented in light colours indicate circa-littoral habitats and dark grey deep-sea habitats. Habitat codes: A6 “Deep-sea bed”; A6.1 “Deep-sea rock and artificial hard substrata”; A6.3 “Deep-sea sand”; A6.4 “Deep-sea muddy sand”; A6.5 “Deep-sea mud”; A6.81 “Canyons, channels, slope failures and slumps on the continental slope”; A5.25 “Circalittoral fine sand”; A5.26 “Circalittoral muddy sand”; A5.27 “Deep circalittoral sand”; A5.35 “Circalittoral sandy mud”; A5.37 “Deep circalittoral mud”; A5.44 “Circalittoral mixed sediments”; A5.45 “Deep circalittoral mixed sediments”.

We found a good relation between the habitats and depths of exploitation by crustacean trawlers and the habitats of occurrence of the main targeted species at the different regions of Portugal (Fig. 2). Hence, in the North-West and Central-West regions, shallow circa-littoral habitats are more exploited in order to capture fish and cephalopods, while in the South-West and South coasts, deep-sea habitats of mud and muddy-sand are frequently targeted for crustaceans (Fig. 2). In the North and Central-West trawling occurs in waters shallower than 200 m, while in the South-West and South we estimated that approximately 75% of the time, trawling was carried out in waters deeper than 200 m, with highest intensities occurring between 100 and 700 m.

## Discussion

This work quantitatively describes the distribution of the trawling effort made by crustacean trawlers and characterizes the most impacted habitats in the Portuguese continental margin using a vessel monitoring system (VMS). To achieve this goal we used some state-of-the-art methodology for the interpolation of positions and speeds of the vessels, as well as for overlapping the geographical information on habitat types and fishing pressure. The tool “vmsbase”^18^ was recently described as a protocol to manage VMS data but given the need to quantify the fishing effort delivered into each port in order to calculate LPUE values, we opted for developing our own code (see Methods). The results shown here are valuable to establish a reference tag for the state of the current bottom trawling fisheries, allowing the analysis of future trends. Additionally, our results present a measure of the current pressure exerted on the benthic marine habitats. Although we have not carried out direct measurements of habitat impact itself, our estimations of trawling effort can be used as a proxy for the degree of disturbance of the habitats.

The distribution of the fishing effort of crustacean trawlers on the Portuguese margin shows clear regional patterns related to the exploited habitats, depths, and species captured (Figs 1 and 2). In the North and Central-West, trawling occurs mostly in shallow circa-littoral habitats, with landings mainly consisting on cephalopods and fish. In contrast, on the South and South-West coasts, trawling is carried out mostly between 100 and 700 m, on muddy and muddy-sand sediments, with main landings of crustaceans in the South and *M. poutassou* and *M. merluccius* in the South-West (Fig. 2). Concomitantly, the main landed species by crustacean trawlers were the rose shrimp and Norway lobster, contributing with more than half of the total biomass landed, followed by hake and common octopus. The total fishing effort by the crustacean trawlers fleet, composed of 25–26 licensed vessels, decreased substantially from 2012 (69596 h y^−1^) to 2014 (63427 h y^−1^), being consistently higher in the South and South-West compared to the North and Central-West regions. The same happened with the landings, passing from 1361 t in 2012 to 992 t in 2014. The decrease in the landings by this fleet, however, could be a longer-term pattern, since in 1998–1999, Afonso-Dias, *et al*.^19^ reported values of 1600 and 2700 t. The allocation of a higher effort in the South and South-West areas in conjunction with the overall decrease in trawling activity is likely a consequence of the observed economic downturn in Portugal in recent years, also referred by Vieira, *et al*.^20^. In fact, the exploitation of the highly valued and more profitable crustacean species may at least partially balance increasing costs of the activity (e.g. fuel) and/or decreasing the time spent at sea.

Considering the trawling effort mentioned above and the landings estimated for this *métier*, we have detected a slight decrease in LPUE values from 2012 (21.65 kg h^−1^) to 2014 (16.40 kg h^−1^). As far as we are aware, the unique estimate of LPUE for Portuguese crustacean trawlers can be found in the work of Afonso-Dias, *et al*.^19^. These authors reported different LPUE of the main target species at the South of Portugal during 1998 and 1999, with values ranging between 1.6 and 5.3 kg h^−1^ for *N. norvegicus*; between 1.2 and 13 kg h^−1^ for *A. antennatus*; and between 10 and 95 kg h^−1^ for *P. longirostris*. In the Blanes region, catches per unit effort of *A. antennatus* reported by Almeida, *et al*. (*in press*)^21^ ranged usually between 5 and 12 kg h^−1^ with peaks reaching 26.7 kg h^−1^ (data from 2002–2004). However, in comparison to previous estimates of LPUE by Portuguese fin-fish trawlers ranging from 14 to 2128 kg h^−1^ (e.g. Gamito, *et al*.^22^, and Pilar-Fonseca, *et al*.^23^), our values seem to be low. Lower LPUE values for crustacean fisheries than for fin-fish are in fact expected, and the overall decrease observed from 2012 to 2014 is also in line with the displacement of effort towards the south (Fig. 1) where the crustaceans are the main target species. Besides the effectiveness and specificity of this *métier* in relation to the available resource, these lower values detected in comparison to other works can also arise from other factors (acting individually or in combination) such as underestimated or unreported landings and unreported discards^24^.

Another problem faced during this work, possibly adding noise to our results, was the non-homogeneity of nomenclature of gears among different Portuguese public data sets (VMS and landings). In these data sets crustacean trawlers are considered in different ways: separately in the VMS data set and together with fin-fish trawlers in the landings reports. We believe that it is of utmost importance to improve the official reporting protocols in Portugal, by standardizing the classification of the *métiers* in every public data set. This will facilitate crossing the information between the data sets and consequently provide more reliable results, which will optimize public expenses.

**Figure 3:**
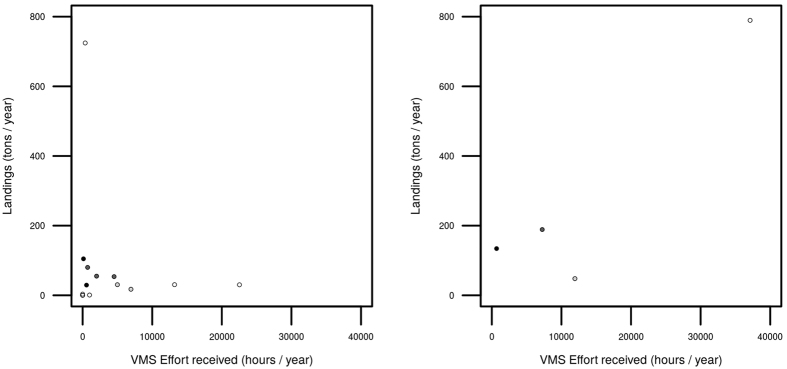
Relationship between the landings and the estimated VMS effort by crustacean trawlers for: the different ports of Portugal (left panel), and the different geographic areas considered (right panel). Black points represent the North-West, dark grey points represent the Central-West, pale grey points represent the South-West, and white points represent the South.

In this study we observed a disparity between the allocation of effort to the different ports from VMS data and respective landings. Contrary to what was expected, no correlation between the trawling effort allocated and the tons of fish landed at each port was observed (Fig. 3a). Here, we need to consider social and economic factors that can directly affect the declared landings at a certain port in a combination of market prices and agreements between ports/countries. Official reports on fisheries of the National Institute of Statistics of Portugal^12^ declares highly variable selling prices for the same species among different ports. In particular, most crustacean species sold in 2014 in VRSA reached 14 €/kg, while in Portimão they only reached 6 €/kg. The proximity of VRSA to the border with Spain, facilitates Spanish commercials to buy crustaceans such as shrimps and lobsters at lower prices than in Spain, with still profitable prices (including transportation). As the demand increases the commercial value increases in comparison to other Portuguese ports, justifying the high landings of crustaceans caught in the South and South-West regions and transported by traffic road to VRSA, where they are computed as landed for the statistics. Similarly, the high landings of blue whiting (*M. poutassou*) at the port of Sines (South-West region) are not related to the geographical proximity of the species habitat and is apparently driven by economic factors, as the fishermen of the port of Sines reached an agreement with Spanish companies in 2010 to sell blue whiting to Spanish sellers at better price than other ports of Portugal, which could cause an increase in the road traffic of fish towards this port. Moreover, the bad correlation shown in Fig. 3a is expectable given that in Portugal, the fish is not necessarily computed in the port of reception but in the port where it is sold. With the aim of minimizing the effects of this practice, we have aggregated the VMS effort and the landings of the different regions (Fig. 3b). In this case the relationship improves considerably, as we expect that the traffic road between different geographic regions is lower than within each region.

Literature on the impact of trawling on deep-sea habitats is still scarce (but see Clarck, *et al*.^6^ and references therein^25^). Morato, *et al*.^26^ showed the existence of a global 50-years trend of trawling deeper as the stocks get over-exploited or depleted in shallow waters. For the North Atlantic, these authors estimated a rate of increasing depth of 32.05 m by year during the last decades. In the Portuguese margin, where crustacean trawling takes place mostly between 100 and 700 m over muddy or muddy-sand bottoms, such increase was not noticeable. Instead, we have found that the effort is more concentrated around 200 and 400 m, albeit the small depth range and timescale may not allow observing such tendencies. Additionally, muddy or muddy-sand bottoms are the habitats of the main landed species (*P. longirostris, A. antennatus* and *N. norvegicus*^27^), so the increase of fishing effort made in this depth range and habitats could indicate a specialization of the effort to capture these species.

During manned submersible dives to the Portimão Canyon, Morais, *et al*.^28^ observed extended areas severely impacted by trawling, suggesting differences in the community composition as a direct consequence of the intense physical disturbance caused by the bottom gears. Later, Fonseca, *et al*.^29^, reported the occurrence of a wide bed of the crinoid *Leptometra celtica*, at approximately 500 m depth, off the Portuguese southern coast located in an enclave of gravelly sand surrounded by muddy sediments. There they found suitable habitats for the Norway lobsters under intense exploitation by the bottom trawling fisheries. The same authors also detected that intensively towed areas present a much lower number of epibenthic fauna in comparison to non-disturbed areas where crinoids were found in high abundances. The marked change in species composition and, potentially in biomass, may suggest the direct or indirect impacts of bottom trawling in the Portuguese southern region. An ongoing study on the impact of crustacean trawling off Sines also points to relevant alterations of the seabed integrity and community structure of megafaunal assemblages^30^. Our data does not allow to provide further conclusions, but other questions arise. For example, the effective extension to which bottom trawling affects the integrity of deep-water habitats and associated fauna remains unclear. Fishing ground areas in the southern Portuguese margin can also harbor large biodiversity hotspots, for example of crinoids, sponges and coral aggregations that we might not yet be fully aware^28^,^29^. This should be of particular concern and subject of future studies, to ensure its representation in non-impacted areas and, for instance, support the establishment of deep-water Marine Protected Areas.

Lastly, it is important to consider that the effects of trawling on the ecosystem are not only direct^6^. Trawling is not a selective gear, and so many other species are captured as by-catch, after which can be discarded or landed. The discards can be composed by non-commercial species, by individuals that do not reach the legal size to be fished (as is the case of 21% of the landed weight of hake, according to the Scientific Report of the Commission of the European Communities^31^), or by species with negligible commercial value. In the case of the Portuguese trawling fisheries in the southern region, discarding can reach up to 70% of the total catch weight^32^,^33^, with more than 140 different species registered^33^, such as sharks (e.g. *Scyliorhinus canicula*), blue withing and monkfish^27^. It is conceivable that changes in seabed, promoted by the bottom trawling gears, might enhance changes in community composition and structure. Furthermore, by-catch and discards of non-targeted species may have consequences for deep-water communities by disrupting trophic structure and changes in intra and interspecific interactions. Therefore, the extent of these impacts should be adequately evaluated in order to understand consequences for diversity and function, and disturbance of biogeochemical cycles.

In conclusion, we have shown that the distribution of the effort by crustacean trawlers in Portugal is mostly concentrated in the southern regions, at depths between 100 and 700 m on muddy and muddy-sand habitats. This activity is currently widely dispersed geographically, impacting more than 10,000 km^2^ per year, essentially in the search of rose shrimp and Norway lobster. The importance of this activity in the North and Central coasts, however, is much more reduced, occurring at shallower waters and targeting fish and cephalopods. During the three years of study (2012, 2013, and 2014) few changes can be appreciated, albeit it seems that some displacement of the effort from the north to the southern regions has happened. We have also found a mismatch between the VMS effort received by each port and the fish landings registered, which seems to be related to economic or social factors such as road communications, or international relationships. The results presented here provide new data on western Iberian Margin ecosystems and represent a scientific evidence of human-induced disturbances on the deep-sea, information of relevance in order to effectively achieve the goals stated on the Marine Strategy Framework Directive^34^.

## Methods

### Effort estimation

To estimate the trawling effort of crustacean trawlers we used data from the VMS of Portugal provided by the Direção Geral de Recursos Naturais, Segurança e Serviços Marinhos (DGRM). The VMS reports the position and velocity of crustacean trawlers longer than 15 m at time steps of 10 minutes within 6 nautical miles from the coast, and of 2 h when trawlers are located farther than 6 nautical miles. Ten trawling vessels were randomly chosen from the universe of licensed crustacean trawlers (26 in 2012, and 25 vessels in 2013 and 2014), and their trajectories, together with the assignment of fishing effort received by each port (see below) were analyzed using R^35^.

**Figure 4:**
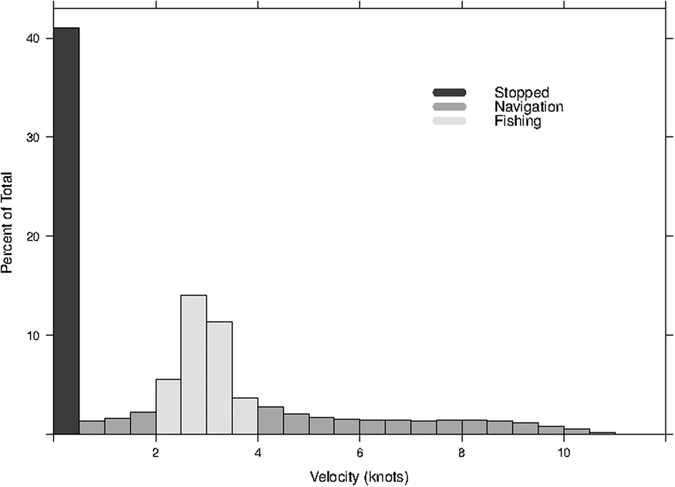
Distribution of velocities (%) for the 10 crustacean trawlers analysed in 2012, showing the range of velocities used to define where fishing took place.

The R code first approached the raw data by deleting incomplete or duplicated registers. Then, an interpolation of the position and velocity of the vessels was performed to every minute of the year using a cubic Hermite spline function^36^,^37^. During the interpolation of positions, some artifacts such as vessels crossing land areas could be caused. Other problem was the definition of erroneous trawling areas when the interpolation calculated fishing speeds between two pings out of the range determined for fishing (see definition of trawling points below). To correct these artifacts, we defined a variable “status” of the vessel which was updated every time step (minute). This variable had the levels: “at port”, “navigation”, “land-navigation”, “fishing”, and “fake-fishing”. Since no data from log-books were available, the areas where fishing took place were estimated using the velocity histogram of the vessels (e.g. Witt, *et al*.^38^; Fig. 4). The status “fishing” was hence defined for velocities between 2 and 4 knots (Fig. 4). The status “fake-fishing” was caused by interpolation of velocities in the range of the fishing velocities during short periods of time. To correct this, we disregarded fishing events shorter than 5 minutes, and longer than 4 hours if they were caused by interpolations between registers separated for more than 4 hours. Finally the areas where the vessel’s status was “land-navigation” were removed from the analysis and these trips disregarded.

For each vessel we then defined the number of fishing trips per year, the duration of each trip, the proportion of trawling time, the origin and landing port, and the time spent at each port. The estimation of the total crustacean trawling fleet effort was made by extrapolating the total effort calculated for the ten analyzed vessels to the total number of registered crustacean trawlers in Portugal for each year. Finally, given that the landing port of each fishing trip was known, the estimations of effort were distributed among the ports where the vessels arrived, assigning to each port a number of trawling hours received per year.

### Impact index

As a measure of the impact caused by trawling, we calculated the time spent trawling per year in cells of 0.1 decimal degrees along the Portuguese continental margin. This information allows us to compare the pressure exerted by trawlers in different regions and years. Similarly, considering the effective trawling speed (average 3 knots) and that the opening of the trawling doors is 80 m (the same assumed by Oberle *et al*.^39^ for Galician trawlers), we can estimate the percentage area of the geographic cells that is trawled in a year (see the work of Watling and Norse^40^), considering that each cell of 0.1 × 0.1 decimal degrees corresponds to 1.006 km^2^.

### Habitat and depth characterization

Using the coordinates of the points where trawling occurred, we estimated the time spent trawling at each habitat and water depth. Information on benthic habitats and bathymetry was obtained from the European Marine Observation and Data Network (EMODNET) seabed habitat and bathymetry portals respectively. Additionally, data from the UNEP’s Global Seafloor Geomorphic Features Map (GSGFM)^41^ was taken into account to cover deep-sea areas that were not available in the EMODNET seabed habitats portal. Habitats from the EMODNET portal were classified following the hierarchical habitat classification system from the European Union Nature Information System (EUNIS)^42^. The geomorphologic features of the seabed (GSGFM) were transformed to the EUNIS classification system following the work of Tempera, *et al*.^43^: escarpments were classified as “A6.1: Deep-sea rock and artificial hard substrata”, canyons as “A6:81: Canyons, channels, slope failures and slumps on the continental slope”, and Abyssal plains as “A6:5: Deep sea mud”. Overlap of coordinates with corresponding bathymetric profiles and habitat maps was performed using the package “sp” of R and spatial analysis tools of ArcGIS v.10.2.

### Assessment of landings from Portuguese crustacean trawlers

Public data on landings at the Portuguese ports were provided by the DGRM. This data set reports yearly total landed biomass (kg) of each species by the port of landing, and the gear deployed considering three categories: multi-gear, purse seine and trawl. In order to cross it with our estimates of effort, and given that the VMS data was divided in crustacean and fin-fish trawlers, we estimated the proportion of landings captured by crustacean trawlers following the work of Campos, *et al*.^11^, who defined different fleet components within the trawling *métiers*. This classification is based on target species and composition of catches, assigning the respective proportions of trawling landings into crustacean and fin-fish trawlers for Portugal. Hence, we ascribed the corresponding proportion of landings to crustacean trawlers for the crustaceans *Parapenaeus longirostris* and *Nephrops norvegicus*, the cephalopods Octopodidae and *Sepia officinalis*, and the fishes *Trachurus trachurus, Scomber scombrus, Micromesistius poutassou, Trisopterus luscus, Merluccius merluccius, Scomber japonicus*. The proportion of these species in the landings reported by crustacean trawlers is provided by Campos *et al*.^11^. According to these authors, these species represent 74.8% of the total landings by the fleet segments assigned to crustacean trawlers. On the other hand, for the species that were not included in the work of Campos, *et al*.^11^ (25.2% of the trawling landings), given that their proportion in the landings by crustacean trawlers was unknown, we assumed that all the fish species were captured by fin-fish trawlers and that crustaceans were captured by crustacean trawlers. The bivalves and gastropods captured by trawlers (which represent 0.16% of the landings) were excluded from our landings estimates. In the case of the cephalopods not included in Campos, *et al*.^11^, we considered the main habitat of each species (following the work of Torres, *et al*.^44^, assuming that benthic cephalopods (e.g. *Alloteuthis* spp., *Eledone cirhosa, Sepia elegans*) were captured by crustacean trawlers while benthopelagic cephalopods (e.g. *Loligo* spp., *Sepietta* spp., *Sepiola* spp., *Todarodes sagittatus*) were captured by fin-fish trawlers. No data on landings of Portuguese trawlers in Spain were available, which can lead to an underestimation of the LPUE (kg of landed fish per trawling hour). LPUE values were calculated by dividing the total estimated landings in Continental Portugal (kg y^−1^) by the total estimated fishing effort of the crustacean fleets (trawling h y^−1^).

### Definition of geographic regions

The Portuguese margin was divided into four main geographic regions with the purpose of identifying geographic patterns for crustacean trawlers activity. The North-West coast comprises the area between the Minho river and the estuary of Aveiro (40.62 to 41.86 N), containing the ports of Viana do Castelo, Matosinhos, and Aveiro. The Central-West area starts south of Aveiro and extends until Cabo da Rocha (38.77 to 40.62 N), with the ports of Figueira da Foz, Nazaré and Peniche. The South-West region comprises the ports of Sesimbra and Sines, and goes from Cabo da Rocha until the Sagres Canyon (37.01 to 38.77 N). Finally, the South region comprises the south-facing coast from the Sagres Canyon to the Guadiana river (35.7 to 37.01 N), comprising the ports of Sagres, Lagos, Portimão, Quarteira, Olhão, and VRSA.

## Additional Information

**How to cite this article**: Bueno-Pardo, J. *et al*. Deep-sea crustacean trawling fisheries in Portugal: quantification of effort and assessment of landings per unit effort using a Vessel Monitoring System (VMS). *Sci. Rep.* **7**, 40795; doi: 10.1038/srep40795 (2017).

**Publisher's note:** Springer Nature remains neutral with regard to jurisdictional claims in published maps and institutional affiliations.

## Figures and Tables

**Table 1:** Average trawling time (h y^−1^) and mean cumulative percentage of the area of the geographic cells trawled during 2012, 2013, and 2014, considering the different geographic regions of the Portuguese coast.

Region	2012 h y^−1^	%	2013 h y^−1^	%	2014 h y^−1^ e	%
North-West	1.82	80	0.88	39	0.69	30
Central-West	2.36	104	1.78	79	1.74	77
South-West	5.13	226	3.91	173	5.84	258
South	11.93	527	11.21	495	10.26	453

Only the impacted cells were considered to calculate these averages.

**Table 2:** Distribution of the trawling effort (h y^−1^) and their relative contribution (%) by region and landing port based on estimated VMS data for 2012, 2013, and 2014.

Region	Port	2012 h y^−1^	%	2013 h y^−1^	%	2014 h y^−1^	%
North-West	Viana do Castelo	—	—	—	—	3	<0.01
	Matosinhos	1024	1.5	390	0.7	264	0.5
	Aveiro	206	0.3	130	0.2	30	0.05
	**Total region**	**1230**	**1**.**8**	**520**	**0**.**9**	**297**	**0**.**56**
Central-West	Figueira da Foz	5979	9.1	3949	7	3647	7
	Nazaré	873	1.3	1177	2.2	101	0.2
	Peniche	1068	1.6	2679	5.0	2267	4.3
	**Total region**	**7920**	**12**	**7805**	**14**.**2**	**6015**	**11**.**5**
South-West	Sesimbra	4432	6.7	4047	7.5	6514	12.5
	Sines	7459	11.4	5228	9.8	8139	15.7
	**Total region**	**11891**	**18**.**1**	**9275**	**16**.**3**	**14653**	**28**.**2**
South	Olhão	16327	24.9	13639	25.7	9671	18.6
	Portimão	27142	41.4	20265	37.8	20196	38.9
	Quarteira	—	—	—	—	—	—
	Sagres	1023	1.5	1035	1.9	924	1.8
	Lagos	—	—	—	—	—	—
	VRSA	—	—	958	1.8	154	0.3
	**Total region**	**44492**	**67**.**8**	**35897**	**67**.**2**	**30945**	**59**.**6**
**Total**		**65533**	**100**	**53498**	**100**	**51910**	**100**

The mismatch between the total effort received by Portuguese ports and the trawl effort calculated in Portuguese waters is caused by a part of the effort being delivered in Spanish ports.

**Table 3:** Distribution of the crustaceans trawlers landings (t y^−1^) and their relative contribution (%) by each Portuguese regions and ports for 2012, 2013, and 2014.

Region	Port	2012 t y^−1^	%	2013 t y^−1^	%	2014 t y^−1^	%
North-West	Viana do Castelo	—	—	—	—	—	—
	Matosinhos	32.22	2.36	33.84	3.00	21.92	2.20
	Aveiro	125.7	9.23	107.85	9.56	80.95	8.15
	**Total region**	**157**.**92**	**11**.**59**	**141**.**69**	**12**.**56**	**102**.**87**	**10**.**35**
Central-West	Figueira da Foz	63.86	4.69	51.32	4.55	45.90	4.62
	Nazaré	84.12	6.17	93.35	8.27	62.83	6.33
	Peniche	57.57	4.23	60.93	5.40	46.69	4.70
	**Total region**	**205**.**55**	**15**.**09**	**205**.**6**	**18**.**22**	**155**.**42**	**15**.**65**
South-West	Sesimbra	26.79	1.96	28.71	2.54	36.00	3.62
	Sines	22.56	1.65	13.64	1.21	16.06	1.61
	**Total region**	**49**.**35**	**3**.**61**	**42**.**35**	**3**.**75**	**52**.**06**	**5**.**23**
South	Olhão	22.26	1.63	40.71	3.61	28.62	2.88
	Portimão	32.09	2.35	31.59	2.80	27.02	2.72
	Quarteira	3.23	0.23	4.94	0.43	1.96	0.19
	Sagres	0.12	0.009	2.28	0.20	0.002	<0.01
	Lagos	0.11	<0.01	—	—	—	—
	VRSA	890.55	65.42	658.54	58.39	624.49	62.92
	**Total region**	**948**.**36**	**69**.**64**	**738**.**06**	**65**.**43**	**682**.**09**	**68**.**71**
**Total**		**1361**.**18**	**100**	**1127**.**7**	**100**	**992**.**44**	**100**
